# Analytical treatment interruption among women with HIV in southern Africa who received VRC01 or placebo in the Antibody Mediated Prevention Study: ATI stakeholder engagement, implementation and early clinical data

**DOI:** 10.1002/jia2.26495

**Published:** 2025-06-03

**Authors:** Shelly Karuna, Fatima Laher, Sufia Dadabhai, Pei‐Chun Yu, Doug Grove, Catherine Orrell, Joseph Makhema, Mina C. Hosseinipour, Carrie‐Anne Mathew, William Brumskine, Nyaradzo Mgodi, Philip Andrew, Lucio Gama, Carissa Karg, Gail Broder, Kagisho Baepanye, Jonathan Lucas, Michele Andrasik, Simbarashe Takuva, Manuel Villaran, Azwidihwi Takalani, Randall Tressler, Lydia Soto‐Torres, Amanda S. Woodward Davis, Ames Dhai, Ian M. Sanne, Myron S. Cohen, Lawrence Corey, Glenda Gray, Allan C. deCamp, Katharine J. Bar

**Affiliations:** ^1^ Vaccine and Infectious Disease Division Fred Hutchinson Cancer Center Seattle Washington USA; ^2^ Perinatal HIV Research Unit Faculty of Health Sciences University of the Witwatersrand Johannesburg South Africa; ^3^ Department of Epidemiology Johns Hopkins Bloomberg School of Public Health Blantyre Malawi; ^4^ Desmond Tutu HIV Centre Institute of Infectious Disease and Molecular Medicine & Department of Medicine University of Cape Town Cape Town South Africa; ^5^ Botswana Harvard AIDS Institute Partnership Gaborone Botswana; ^6^ Department of Immunology and Infectious Diseases Harvard T.H. Chan School of Public Health Boston Massachusetts USA; ^7^ Department of Medicine University of North Carolina at Chapel Hill Chapel Hill North Carolina USA; ^8^ UNC Project Malawi Lilongwe Malawi; ^9^ Wits RHI University of the Witwatersrand Johannesburg South Africa; ^10^ The Aurum Institute NPC Johannesburg South Africa; ^11^ Department of Medicine School of Medicine Vanderbilt University Nashville Tennessee USA; ^12^ Clinical Trials Research Centre University of Zimbabwe College of Health Sciences Harare Zimbabwe; ^13^ FHI 360 Durham North Carolina USA; ^14^ Vaccine Research Center National Institute of Health Bethesda Maryland USA; ^15^ School of Health Systems and Public Health Faculty of Health Sciences University of Pretoria Pretoria South Africa; ^16^ Chris Hani Baragwanath Academic Hospital Soweto South Africa; ^17^ Department of Family Medicine and Primary Care Faculty of Health Sciences University of Witwatersrand Johannesburg South Africa; ^18^ National Institutes of Health Bethesda Maryland USA; ^19^ Division of AIDS National Institute of Allergy and Infectious Diseases National Institutes of Health Bethesda Maryland USA; ^20^ School of Clinical Medicine University of the Witwatersrand Johannesburg South Africa; ^21^ South African Medical Research Council Johannesburg South Africa; ^22^ Clinical HIV Research Unit Faculty of Health Sciences University of the Witwatersrand Johannesburg South Africa; ^23^ Institute for Global Health and Infectious Diseases The University of North Carolina at Chapel Hill Chapel Hill North Carolina USA; ^24^ Department of Laboratory Medicine University of Washington Seattle Washington USA; ^25^ South African Medical Research Council Cape Town South Africa; ^26^ Perelman School of Medicine University of Pennsylvania Philadelphia Pennsylvania USA

**Keywords:** ATI, HIV remission, broadly neutralizing antibodies, stakeholder engagement, HIV cure, Africa

## Abstract

**Introduction:**

Antiretroviral therapy (ART) prevents and treats, but does not eradicate, HIV. Early ART initiation is associated with post‐ART virologic control, particularly among African women, and anti‐HIV‐1 broadly neutralizing antibodies (bnAbs) may modulate immune responses to HIV. We evaluate whether early ART with or without anti‐HIV‐1 bnAb VRC01, present at HIV acquisition, is associated with later ART‐free control in African women and we assess potential associations with observed control.

**Methods:**

Stakeholder engagement informed analytical treatment interruption (ATI) study design and implementation. Participants who received placebo or VRC01 and acquired HIV in the Antibody Mediated Prevention efficacy trial were assessed for ATI eligibility, including HIV acquisition within 8 weeks of receiving VRC01 or placebo, followed by early ART initiation and ≥1 year of viral suppression. Participation facilitators and barriers were assessed. From May 2021 to February 2024, participants enrolled, stopped ART and received frequent viral load and CD4+ T‐cell count monitoring for safety and assessment of meeting ART reinitiation criteria.

**Results:**

Thirteen women enrolled from southern Africa. No ATI‐related serious adverse events (AEs), HIV transmissions, pregnancies or ≥Grade 2 AEs were observed. Eight sexually transmitted infections were diagnosed in seven women during ATI. Two participants had tenofovir levels consistent with use during ATI; 2/11 (18%) who completed ATI without antiretroviral use exhibited ART‐free control for ≥32 weeks. The median time to confirmed VL≥200 was 5.4 weeks (range 2.7−112). The most common ART reinitiation criterion met was virologic (*n* = 7). VRC01 receipt proximate to HIV acquisition was not associated with control. Controllers versus non‐controllers did not differ by early post‐acquisition viral load kinetics, acquired virus characteristics, or time from estimated acquisition to closest infusion or to ART initiation.

**Conclusions:**

In a safe, well‐tolerated ATI, 18% of 11 African women exhibited post‐intervention control. Design and implementation lessons inform future ATIs in Africa. Analyses of peri‐acquisition and post‐ATI host and viral characteristics can inform the development of interventions for HIV cure, prevention and treatment.

**Clinical Trial Registration:**

NCT04860323

## 1 INTRODUCTION

In most people with HIV (PWH), modern antiretroviral therapy (ART) enables prolonged suppression of viremia and improves lifespan and quality of life. For many, however, ART incurs long‐term toxicity, drug−drug interactions, stigma, drug resistance, adherence challenges and pill fatigue. Furthermore, ART is unable to eradicate HIV; upon cessation of therapy, viremia rebounds rapidly with the reactivation of persistent viral reservoirs in almost all PWH [1, 2, 3, 4].

Immunotherapeutic alternatives to ART are a high priority in HIV research and have shown promise. HIV‐specific broadly neutralizing antibodies (bnAbs), present at the time of ART initiation, may amplify the immune system's capacity to contribute to ART‐free control [5, 6]. BnAbs suppress viremia [5, 7, 8, 9, 10], modulate the autologous immune response to acute HIV infection [10, 11, 12, 13, 14, 15, 16, 17, 18, 19, 20, 21, 22, 23, 24, 25, 26, 27], and may limit the establishment and decrease the size of the viral reservoir [12, 28, 29, 30, 31, 32, 33, 34, 35]. Early ART initiation has also been associated with delayed time to rebound and increased frequency of ART‐free virologic control in “post‐treatment controllers” [28, 36, 37].

These associations of early ART initiation with delayed rebound and post‐treatment control (PTC) have been particularly notable in analytical treatment interruptions (ATIs) conducted among southern African women with HIV, up to 25% of whom have remained virally suppressed in ATIs for >12 weeks [38] and up to a median of 4.5 years [39]. In contrast, 0–6.4% of individuals exhibited control in largely male, American cohorts [28, 39].

African women who initiate ART early in disease appear to have uniquely high rates of later ART‐free virologic control, which could inform broader efforts to achieve ART‐free control. Furthermore, women in southern Africa are among the most heavily burdened by HIV globally and African research communities have called for authentic inclusion and co‐leadership of the HIV cure research agenda [40, 41, 42, 43, 44, 45, 46, 47].

We conducted an ATI among former participants of the HVTN 703/HPTN 081 Antibody Mediated Prevention (AMP) HIV prevention efficacy study in Africa to evaluate the potential impact of early ART initiation with or without bnAbs received near the time of HIV acquisition among women in southern Africa. In addition to early ART initiation, AMP participants randomized to receive VRC01 had circulating bnAb at the time of HIV acquisition, enabling the potential formation of immune complexes of VRC01 bound to free virions or HIV‐infected cells, which may modulate the immune response and, thus, the course of HIV [21, 22]. Therefore, this cohort offers a singular opportunity to assess the potential impact of early ART initiation and very early immunotherapy on post‐acquisition immunologic and virologic trajectories following HIV acquisition. We share key design, development and implementation considerations, and safety and clinical outcomes from this ATI, the first—but not the last [48]—conducted in the current era among African women with HIV.

## 2 METHODS

### 2.1 The AMP Study and conceptualization of a post‐AMP ATI

The HVTN 703/HPTN 081 AMP Study, conducted from May 2016 to November 2020, was the parent trial to the HVTN 805/HPTN 093/A5393 post‐AMP ATI reported here. The AMP Study enrolled 1924 women without HIV to evaluate the HIV prevention efficacy of the VRC01 bnAb; participants were randomized to receive 10 or 30 mg/kg VRC01 or placebo via intravenous (IV) infusion every 8 weeks over 72 weeks [49, 50, 51].

Participants who acquired HIV were diagnosed shortly after acquisition due to monthly HIV diagnostics; among participants who acquired HIV and initiated ART, the first HIV RNA‐positive sample was identified, retroactively, at a median of 2 weeks before the confirmatory HIV diagnosis. These participants discontinued further study infusions and were monitored closely for 24 weeks post‐diagnosis. The subset of AMP participants who later enrolled in the AMP ATI initiated ART a median of 3 weeks after HIV diagnosis confirmation.

### 2.2 Stakeholder engagement

Clinical trial stakeholder engagement is a necessary component of Good Participatory Practice [52] and crucial for a trial with novel design elements and risk‐benefit considerations for participants, their sexual partners and their communities [40, 41]. The study team included PWH, local Community Advisory Board members, clinical research site (CRS) staff, local investigators and ethicists, and global leaders in HIV remission research. Consultations with this team and additional stakeholders began early in study development and continued throughout implementation. Consultation topics were identified through a literature review and dialogue with HIV remission experts, study team members and CRS representatives. At each consultation's conclusion, stakeholders completed an anonymous evaluation with text fields for detailed feedback, which were reviewed for extraction of common themes (Table 1).

**Table 1:** Baseline characteristics

	Total	Placebo	VRC01 10 mg/kg	VRC01 30 mg/kg
**Total enrolled**	13	6	5	2
**Assigned sex at birth** ^a^				
Female	13 (100.0%)	6 (100.0%)	5 (100.0%)	2 (100.0%)
**Ethnicity**				
Not Hispanic or Latino	13 (100.0%)	6 (100.0%)	5 (100.0%)	2 (100.0%)
**Age (years)** ^b^				
21–30	1 (7.7%)	1 (16.7%)	0 (0.00%)	0 (0.00%)
31–40	12 (92.3%)	5 (83.3%)	5 (100.0%)	2 (100.0%)
Median (min, max)	33 (23−40)	32 (23−35)	35 (31−40)	33 (31−35)
**Race**				
Black	12 (92.3%)	5 (83.3%)	5 (100.0%)	2 (100.0%)
Non‐Black/Mixed	1 (7.7%)	1 (16.7%)	0 (0.00%)	0 (0.00%)
**Country**				
Zimbabwe	4 (30.8%)	3 (50.0%)	0 (0.00%)	1 (50.0%)
Botswana	1 (7.7%)	0 (0.00%)	0 (0.00%)	1 (50.0%)
South Africa	4 (30.8%)	1 (16.7%)	3 (60.0%)	0 (0.00%)
Malawi	4 (30.8%)	2 (33.3%)	2 (40.0%)	0 (0.00%)
**Time (days)**				
Closest AMP infusion to estimated acquisition date, median (min, max)	7 (−22 to 34)	−8 (−16 to 34)	14 (−22 to 17)	10 (7–13)
Estimated acquisition date to ART initiation, median (min, max)	79 (40–147)	80 (77–147)	64 (40–82)	87 (86–88)
Duration of ART^c^	1280 (923–1722)	1326 (923–1722)	1340 (1203–1371)	1260 (1240–1280)

^a^ Gender identity is not assessed for participants outside of South Africa; all participants in South Africa identified as female.

^b^ Age at HVTN 805/HPTN 093/A5393 enrolment date.

^c^ Defined as the time from ART initiation to the start of the ATI.

**Figure 1:**
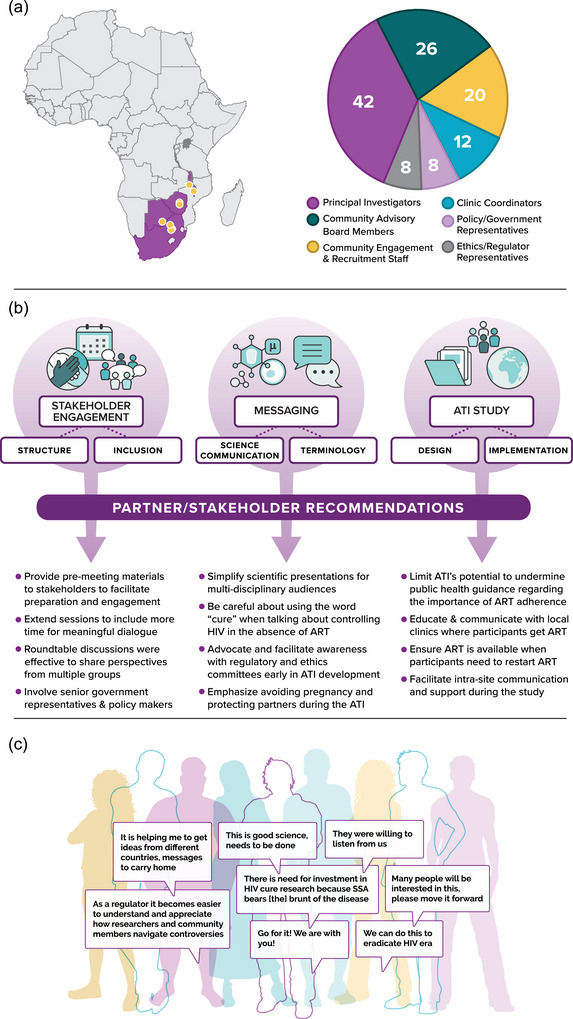
AMP ATI stakeholder engagement. (A) Clinical research sites, countries and roles of AMP ATI stakeholder meeting participants. (B) Themes and recommendations raised in AMP ATI stakeholder engagement prior to AMP ATI Study launch. (C) Quotes excerpted from post‐meeting surveys completed by AMP ATI stakeholder meeting attendees regarding the stakeholder meetings and the AMP ATI Study and HIV remission research that was discussed there.

Multiple steps facilitated in‐person consultation attendance, including holding them in Africa, funding stakeholder attendance, and designing agendas to balance efficiency with sufficient opportunity for discussion and questions. Stakeholder attendees included Ethics Committee and Regulatory Authority representatives, investigators, policy representatives, community engagement representatives and advisors, HIV care providers and local key opinion leaders, including tribal and spiritual leaders (Figure 1A).

Key topics discussed during stakeholder engagement included potential concerns about ATI from regional primary HIV care providers and the need to partner with them; the rationale for the inclusion of AMP placebo recipients; risks of HIV transmission to partners or in pregnancy during ATI; and concerns about potential ART resistance and “stock‐outs” at ART reinitiation.

Stakeholders highlighted the potential impact of an ATI on local public health messages regarding the importance of early ART initiation and adherence. Though this concern was addressed in part by the intentional omission of broad public messaging about the AMP ATI—participants were drawn from an earlier “parent” trial, not recruited from the general PWH population—in screening and throughout informed consent, participants were reminded of the importance of ART adherence and advised that an ATI should not be undertaken outside of close monitoring in a study.

Other concerns raised during stakeholder engagement were also addressed through a robust informed consent process, including an informed consent form (ICF) and related materials developed with community representatives, an animated video that condensed ICF concepts into an accessible format translated into local languages, and an Assessment of Understanding to help ensure participant understanding of the study. The team also employed shared decision‐making, utilizing decision‐making aids and assessments at screening and throughout the ATI. These questionnaires were based on a participant‐led shared decision‐making model validated across healthcare settings, including in other ATIs, to facilitate high‐quality, well‐informed decisions aligned with potential participants’ personal values and preferences [53, 54, 55].

### 2.3 Study design and implementation

The AMP ATI was designed in 2018–2019 and informed by two publications that reflected multistakeholder expertise regarding the conduct of ATIs among PWH: the Treatment Action Group (TAG) community recommendations [56] and the Ragon consensus recommendations [57].

Consensus eligibility criteria were applied in the AMP ATIs, including inclusion of participants with evidence of early ART initiation and ≥1 year of virologic suppression on ART and exclusion of those with evidence of hepatic or renal dysfunction, untreated sexually transmitted infections (STIs), active or latent (LTBI) tuberculosis (unless, for LTBI, ≥1 month of treatment was completed and was ongoing, as applicable), hepatitis C or other significant medical conditions. Enrolment began in May 2021; follow‐up is projected to be complete in 2025.

#### 2.3.1 Placebo group inclusion

The inclusion of participants who received a placebo in the parent AMP Study was extensively considered. Consensus ATI guidance emphasizes scientific veracity—the necessity to ensure that the intentional interruption of ART is uniquely required and well‐designed to answer an important scientific question [56, 57]. The AMP ATI sought to explore whether serum VRC01 circulating at the time of HIV exposure, even if unable to prevent acquisition, might favourably alter the early immune response to HIV, potentially enabling immune‐mediated ART‐free HIV control. All AMP ATI participants initiated ART early, comparable to participants in earlier studies who exhibited control associated with early ART alone; thus, AMP placebo recipients were included in the AMP ATI to limit potential misattribution of virologic control that might be observed among VRC01 recipients and that could be due to early ART initiation alone. Notably, earlier studies’ design elements [38, 39]—including ART reinitiation criteria, monitoring frequency and methods, and eligibility criteria—differed from the AMP ATI, limiting their appropriateness as direct historical controls.

#### 2.3.2 Study procedures

Initial pre‐screening of participants who acquired HIV in AMP was conducted by treatment‐unblinded statisticians, who identified potentially eligible AMP participants based on the proximity of their estimated date of HIV acquisition to their closest AMP infusion (i.e. if the 95% confidence interval around the estimated HIV acquisition date fell within 8 weeks of their closest AMP infusion, see [58]); their ART initiation relative to their estimated HIV acquisition date (i.e. within 24 weeks); and AMP treatment assignment. CRSs conducted further phone pre‐screening ± in‐person screening (i.e. comprehensive eligibility assessment) of pre‐screened potential participants.

**Figure 2:**
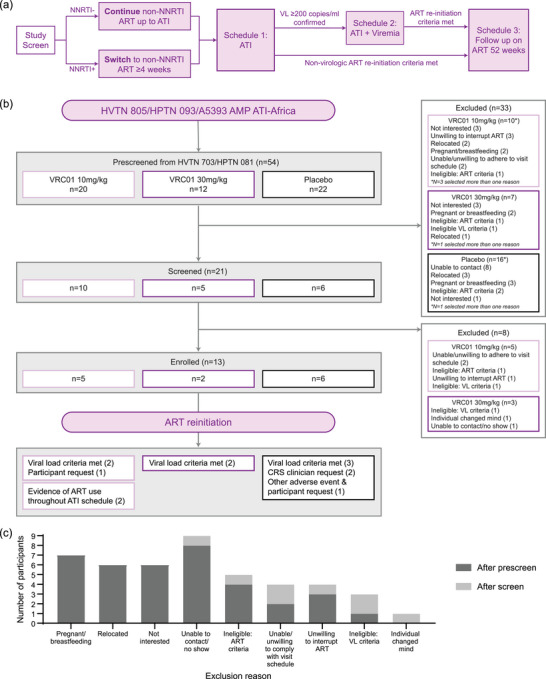
Trial schema. (A) HVTN 805/HPTN 093/A5393 AMP ATI‐Africa design schematic. (B) CONSORT and ART reinitiation criteria. (C) Graph of exclusion reasons after pre‐screening (dark grey) and screening (light grey).

Eligible participants on non‐nucleoside reverse transcriptase inhibitor (NNRTI)‐containing ART regimens at screening underwent a switch to non‐NNRTI‐based ART for at least 4 weeks pre‐ATI. During ATI, participants were initially monitored weekly with viral load (VL) and every other week with CD4+ T‐cell counts. With increasing duration of ART‐free virologic control, monitoring was gradually reduced to every 2–4 weeks, then, after 1 year of ART‐free control, to every 8–12 weeks (Figure 2A, Schema).

A decision aid completed during screening facilitated informed consent and identified enrolment motivators and barriers. Decision assessments were completed throughout the study, including at each visit schedule change, to ensure ongoing understanding and consent. A psychosocial assessment was also conducted during screening and throughout the study to facilitate care for participants’ mental health. The informed consent proffered coverage of emotional and physical study‐related injury, and before study implementation, CRSs identified accessible mental health resources for referral, as needed.

Chlamydia, gonorrhoea, trichomonas and syphilis testing was conducted monthly and as indicated during ATI. Dried blood spot (DBS) and/or plasma samples were stored for antiretroviral testing, which was conducted on samples from participants with viral suppression for ≥12 weeks on ATI. Hematologic, hepatic and renal function was monitored monthly for at least the first 9 months, then every 8–12 weeks, throughout ATI.

#### 2.3.3 Pregnancy and partner protections

Eligibility depended on participants’ commitment to avoid pregnancy throughout the ATI and until virologic re‐suppression after ART reinitiation, including consistent and correct use of a barrier method plus another form of effective contraception. Participants received frequent pregnancy testing, contraception counselling and transmission prevention counselling. Study staff also offered to facilitate STI testing, prevention counselling and pre‐exposure prophylaxis (PrEP) access for participants’ sexual partners, and developed detailed plans describing local PrEP access options.

#### 2.3.4 ART reinitiation

ART reinitiation criteria, aligned with available recommendations [56, 57], reflected the unique scientific questions of the AMP ATI. ART reinitiation criteria included a confirmed CD4+ T‐cell count of <350, HIV‐related symptoms, participant and/or provider request, and sustained viremia >1000 copies/ml for 4 weeks without a 0.5log_10_ decrease. Four weeks of viremia allowed by ART reinitiation criteria balanced safety considerations—limiting prolonged viremia—with the objective of evaluating potential immunologically mediated control.

Upon meeting ART reinitiation criteria, CRSs facilitated ART (re‐)access for rapid restart and re‐suppression, aided by sustained, participant‐permitted communication with ATI participants’ HIV care providers throughout the trial and/or upon ART reinitiation. Though never needed, the study also provided for ART resistance testing if timely viral re‐suppression was not achieved upon reinitiation.

#### 2.3.5 Safety monitoring and laboratory support

Safety oversight was led by HVTN clinicians based in South Africa and familiar with local resources and standards of care, who provided on‐site visits, refresher trainings and real‐time guidance for site questions. Operational support for timely turnaround of safety labs (e.g. VL) was provided by Network laboratory staff who liaised directly with CRSs and endpoint laboratories.

#### 2.3.6 Navigating the unpredictable: COVID‐19

The trial Sponsor, the NIH/NIAID Division of AIDS (DAIDS), granted final approval for the AMP ATI in Africa in May 2020. African regulatory submissions were then intentionally delayed for 6 months as the impact of SARS‐CoV‐2 was assessed and COVID‐19 prevention tools were developed and deployed. These tools included site‐facilitated COVID‐19 vaccine access, SARS‐CoV‐2 testing, private study visit transportation, remote conduct of some procedures to limit in‐person study visit duration, spacing participant visits to limit clinic crowding, and HVTN provision of personal protective equipment to CRSs unable to procure it during significant supply chain disruptions.

#### 2.3.7 Statistical analysis

Nelson−Aalen estimation was used to estimate the cumulative incidence of two endpoints with time origin of ATI initiation: (1) meeting ART reinitiation criteria, and (2) viremia, defined as two consecutive VL measurements ≥200 copies/ml. For endpoint 1, censoring was not necessary as all participants met their endpoint criteria; for endpoint 2, participants were censored at the date of ART reinitiation. Pointwise 95% confidence intervals were reported for cumulative incidence curves and two‐sided 0.05‐level log‐rank tests were used to compare endpoint distributions between AMP VRC01 recipients, pooled across dose groups and placebo recipients.

Parameters of HIV acquisition were compared between non‐controllers and controllers, the latter defined as participants maintaining a confirmed VL <200 copies/ml for ≥24 weeks, using a Wilcoxon rank sum test. Given the number of controllers, *n* = 2, versus non‐controllers, *n* = 9, we have power to detect a difference in a continuous parameter without ties at an alpha level of 0.05 only if there is complete separation between the two groups.

The predicted neutralization 80% inhibitory dilution titre (PT80) measures the neutralization potency of a given antibody serum concentration against a specific HIV‐1 isolate [58]. We computed PT80 from VRC01 concentration at the estimated time of acquisition and neutralization sensitivity of the most VRC01‐sensitive acquired isolate. Correlation was measured using Spearman rank correlation.

## 3 RESULTS

### 3.1 Stakeholder engagement themes

Of 116 stakeholder attendees, 85 (73%) completed evaluations. Attendees described “good discussions” noting that “…all stakeholders present brought interesting discourses” and that “different angles to consider from different groups was very helpful.”

In post‐consultation feedback, three major themes were endorsed: Stakeholder Engagement Structure and Inclusion, the AMP ATI Study's Design and Implementation, and Science Communication and Terminology. Regarding the latter, in the first consultation, held in South Africa in mid‐2019, feedback highlighted the scientific complexity and inaccessibility of some presentations. Stakeholders advised organizers to “simplify the presentations…in a meeting that invites both scientists and community representatives” and observed that it is “hard to mix scientific and community agenda[s]—maybe better to focus on each separately.” Thus, at the South Africa consultation in 2020, community and investigator participants split for several hours to enhance engagement for each group. The success of this was reflected in feedback (e.g. “I [am] confident to…explain about AMP ATI to the community.”) (Figure 1B,C and Table 1).

### 3.2 Screening and enrolment facilitators and barriers

The statisticians identified 53 AMP participants who met ATI pre‐screening eligibility criteria; trial CRSs then assessed additional AMP ATI eligibility criteria and documented reasons for screen failure (i.e. ineligibility or declining further screening, *n* = 40) (Figure 2B). Thirteen participants enrolled from 2021 to 2022. The most common reasons for screen failure included loss to follow‐up, relocation away from the CRS and ongoing or anticipated pregnancy/breastfeeding during ATI (Figure 2C).

All enrolled participants reported that primary facilitators of their decision to participate included careful monitoring of their health and HIV through the study and being able to test their immune system in a “safe environment” and restart their ART at any time. One participant described it as a “privilege to be seen so frequent[ly].” Enrolled participants reported that concerns about participating included the possibility of transmitting HIV to sexual partners and potentially having HIV symptoms, a detectable VL and a drop in CD4 count upon rebound. Similarly, one participant who declined to enrol after screening noted that a key barrier was “the possibility of getting sick [and] making other people in the family and community aware of [her] HIV status.” At baseline, all 13 enrolled participants confirmed that they felt sure about the best choice for them, understood the pros and cons that mattered most to them, and had enough support and information to make the choice. Throughout the trial, all who initiated ATI repeatedly reiterated alignment with their decision to participate.

### 3.3 Demographics

Of the 13 participants enrolled, six received placebo, five received VRC01 10 mg/kg and two received VRC01 30 mg/kg in the HVTN 703/HPTN 081 AMP Study. Reflecting eligibility criteria of this parent AMP Study, all AMP ATI participants were assigned female sex at birth; gender identity was reported only in South Africa, where all identified as women. The median age at enrolment was 33 years (range 23–40). Enrolment was balanced across Malawi (*n* = 4, 30.8%), South Africa (*n* = 4, 30.8%), and Zimbabwe (*n* = 4, 30.8%), and one participant enrolled in Botswana (7.7%) (Table 2).

**Table 2:** Themes and sub‐themes from stakeholder engagements

Subthemes	Quotes^a^
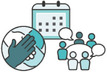	Stakeholder engagement: structure and inclusion
**Pre‐meeting structural changes**	“Provide slides prior to the meeting.”
	“It would have been easier to have questions/assignment to work through and prepare…before the consultative meeting.”
	“Have a session before main session for community only to get them up to speed on basic immunology terms.”
**Meeting agenda and structure**	“Roundtable discussions were an eye opener and very informative.”
	“Open discussion and group roundtable discussion was most helpful.”
	“Discussion of pertinent questions was valuable.”
	“Meeting could have been done over 3 days to avoid ‘data overload’ during sessions [and] have more meaningful dialogue.”
	“Ethical consideration was most helpful.”
	“More time is needed for discussions.”
**Shifting perspectives**	“The meeting was very intriguing and was a mind‐opener for me. Yes we do need…the cure.”
	“An eye‐opening meeting…work hard and let's delete the era of HIV.”
	“I got the chance to appreciate the study even better.”
	“…Unanswered questions answered and fears cleared.”
**Inclusion of multiple stakeholders**	“Allowed key stakeholders to share their concerns constructively.”
	“It was great hearing experiences from different sites.”
	“Inclusionary of all the stakeholders.”
	“Collaboration between scientists and community members was most helpful.”
	“A great workshop and very exceptional…to include community.”
**Government and policymaker involvement**	“Involve the NDOH and other policy makers. It's easier to engage and get community buy‐in if the policy makers are also involved.”
	“…we would like to hear from Department of Health responsibility and government's contribution or ownership in research or entire studies.”
	“Get right and senior representation from local government.”
**Continued stakeholder engagement**	“We need more of these stakeholder consultations…this is quite a complex study.”
	“Community engagement (stakeholder and PHCP) is crucial in this study if it is to be effective.”
	“Suggest that at least more conferences be done for more clarity.”
	“Let us as sites support each other and share our experiences.”

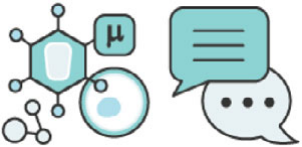	Science communication and terminology
**Simplify the science** ^b^	“There is need for a lexicon of commonly used terms in vernacular languages.”
	“The audience is very multi‐disciplinary…even the basic science talks were…too complex for many.”
	“Simplify the presentations especially in a meeting that invites both scientists and community representatives. Simple language and explanation is paramount.”
	“Hard to mix scientific and community agenda—maybe better to focus on each separately.”
**Clear science** ^c^	“Scientific and detailed presentation—excellent.”
	“The science around interrupting this treatment is well understood.”
	“I could follow the consultation clearly and I feel this would help me as a community educator be able to explain clearly about AMP ATI Study.”
	“I [am] confident to be part of the study and explain about AMP ATI to the community.”
**Use of the word “cure”**	“Look into how we define HIV cure and remission that will not cause high community expectations.”
	“Use the term ‘control of HIV in the absence of ART’ rather than ‘cure’ which can be misleading.”
	“I think we should not [be] quick to say we have found the road for cure since this will bring confusion…”

Subthemes	Quotes
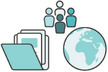	ATI study: design and implementation
**Study design rationale**	“Presentation and discussion surrounding how the interruption has been observed in other studies and how that information has informed the ATI…was most helpful.”
	“Frank discussion on rationale for AMP ATI was most helpful.”
	“Let us involve our participants who were taking part in AMP study irrespective of their arm.”
	“This suggested/envisaged study is very important and relevant.”
**Participant privacy/stigma**	“Maintain privacy as the study is being done…”
	“Suggest researchers [ensure] safety and confidentiality.”
	“Selection of participants unique and retention vital. Avoid stigmatization.”
**Community teams education**	“Develop informative and concise study materials for utilization in the trial.”
	“Education of the community team is vital.”
**Ethics’ committees**	“Let's work on empowering ethics committees.”
	“Literacy and ethics very critical for success of study.”
	“AMP ATI is important for generation of new knowledge. There is a need of advocacy and awareness among REC members.”
**Implementation considerations**	“Consult and discuss with local clinics [where] participants receive treatment from so easier for participant to return to care.”
	“Be careful about participants that may not actually stop treatment when joining the study.”
	“More practical discussions are necessary re: implementation.”

*Note*: Within each of three key themes, subthemes (left column) were identified from quotes (right column) extracted from stakeholder feedback.

^a^ From 72% (61/85) of attendees who completed evaluations at stakeholder consultations, representing all roles reflected in Figure 1A. Due to small numbers of representatives in some subgroups (e.g. see Figure 1A), respondent identifiers are omitted.

^b^ All quotes are from first (2019) stakeholder consultation.

^c^ Last three of four quotes are from second (2020) consultation.

### 3.4 Retention

One participant terminated early upon moving away from the CRS 4 months post‐enrolment; they were virologically suppressed for 14 weeks off ART before resuming ART upon moving. No other early terminations occurred. The final participant initiated her 12‐month post‐ART‐reinitiation follow‐up visit schedule in February 2024. By February 2024, 95% (336/353) of scheduled study visits had been completed overall.

Two of 13 (15%) participants remained on antiretrovirals (ARVs) during their ATI visit schedule without informing their CRS, instances detected during pre‐specified ARV screening. These participants were moved to the ART reinitiation schedule (Schedule 3, Figure 2A) upon detection of ongoing ARV use, and are not included in the primary virologic analyses. All enrolled participants were analysed for safety endpoints.

### 3.5 Safety

Among the 13 enrolled participants, no serious adverse events (SAEs), pregnancies, HIV transmissions or HIV‐related conditions were reported. One AE, a Grade 1 white blood cell count decrease, was deemed related to ATI and resolved spontaneously. Four Grade 3 AEs deemed unrelated to ATI were reported, including one participant with two instances of decreased creatinine clearance, one with increased serum creatinine and one with increased direct bilirubin. Upon meeting ART reinitiation criteria, participants restarted ART within a median of 6.5 days (range 1–23); all were resuppressed within 8 weeks of ART reinitiation.

Eight STIs were diagnosed in seven women during ATI (three diagnoses of gonorrhoea; two syphilis; two trichomoniasis; one genital ulcer syndrome) for a rate of 1.32 STIs per person‐year during ATI. Seventeen STIs were diagnosed in six women after restarting ART while in follow‐up (1.54 STIs per person‐year, overall). Each STI diagnosis was followed by treatment or referral for treatment, as indicated, as well as additional counselling by CRS investigators for further risk mitigation and eligibility re‐assessment.

### 3.6 Time to viremia and ART reinitiation

**Figure 3:**
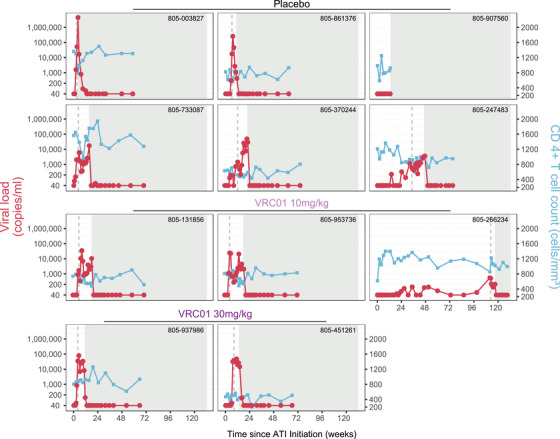
Individual participant viral load and CD4+ T‐cell counts over time during ATI among African women. Viral load shown in red circles, T‐cell counts in blue squares. The treatment each participant received in the parent AMP Study is indicated above each panel. Time of first viremia (i.e. confirmed VL ≥ 200 copies/ml) is indicated by the grey dashed line. Time of meeting ART reinitiation criteria is indicated by the beginning of the grey shaded areas. Two participants with DBS ARV levels consistent with ongoing ARV use during ATI were excluded from this analysis and are not shown here.

Two of 11 (18%) participants who initiated ATI exhibited virologic control (i.e. no confirmed VL ≥200 copies/ml) for ≥32 weeks off ART. One participant (who received VRC01 10 mg/kg in AMP) remained aviremic, with no confirmed VL ≥200, until week 112. This participant's VL remained ≤40 copies/ml through week 19 and between ≤40 and 137 through week 99; then, she had two VL >200 (546 and 514 copies/ml) in week 112 and, though her VL then declined and remained between 174 and 219, she opted to restart ART in week 117. A second participant (who received placebo in AMP) exhibited control for 32 weeks with increases to 223 copies/ml at week 14 and 326 at week 24 before two consecutive VLs ≥200 (1155 at week 32 and 618 at week 34). Her VL then remained 239–1237 copies/ml until week 42, with all but two VLs <1000, before 4 consecutive weeks of VL ≥1000 copies/ml (range 1001–3347), meeting virologic ART reinitiation criteria at week 46 (Figure 3).

**Figure 4:**
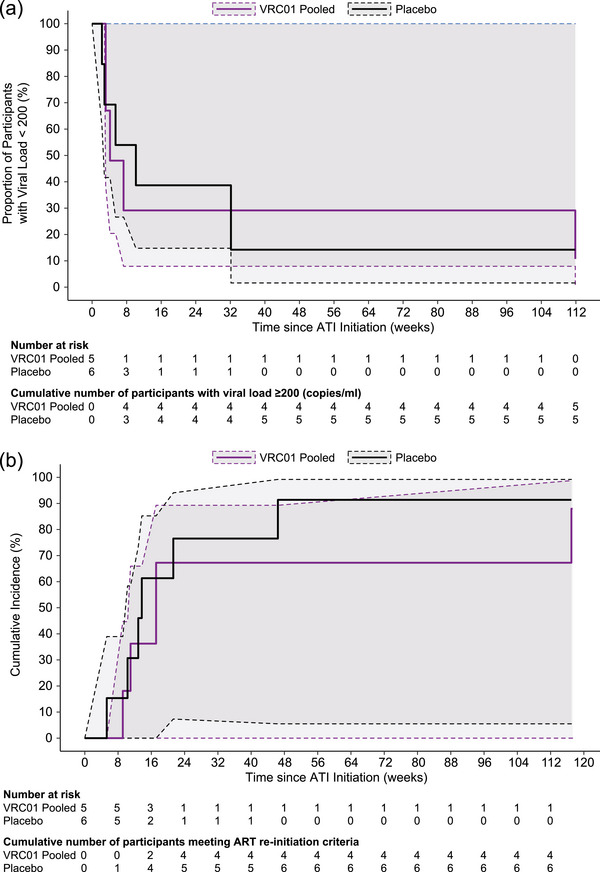
Virologic control by receipt of VRC01 or placebo in the AMP Study. (A) Survival plot of time to viral load ≥ 200 copies/ml. (B) Cumulative incidence of time to meet ART reinitiation criteria. Estimated survival or cumulative incidence is reflected in the solid line and 95% confidence intervals are reflected by the dashed line. Two participants with DBS ARV levels consistent with ongoing ARV use during ATI are excluded from this analysis.

Among those who initiated ATI, no statistically significant difference in time to viremia (Figure 4A; *p* = 0.57) or in time to meet ART reinitiation criteria (Figure 4B; *p* = 0.99) was observed by the AMP treatment assignment. The median time to confirmed VL ≥200 copies/ml was 5.4 weeks overall (*n* = 11), 10.1 weeks among AMP placebo recipients (*n* = 6) and 4.1 weeks among AMP VRC01 recipients (*n* = 5). The median time to meet ART reinitiation criteria was 13.7 weeks overall (*n* = 11), 13.3 weeks among placebo recipients (*n* = 6) and 17.1 weeks among VRC01 recipients (*n* = 5).

Reasons for meeting ART reinitiation criteria included virologic (*n* = 7), CRS clinician request (*n* = 2), participant request (*n* = 2) and AE (*n* = 1, Grade 2 oral herpes simplex infection deemed not related to ATI). Two reasons were provided for one participant—participant request and AE.

**Figure 5:**
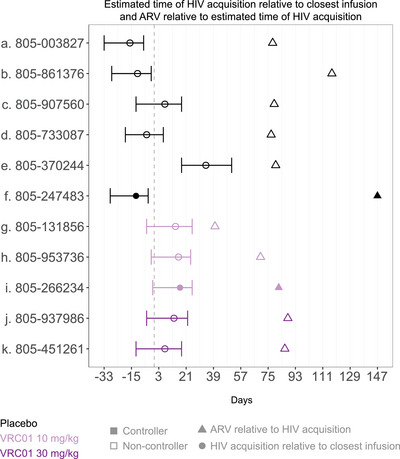
Estimated time of HIV acquisition relative to closest infusion and ART initiation relative to estimated time of HIV acquisition. The vertical dashed line reflects the time of the AMP infusion received closest to the estimated date of HIV acquisition. Two participants with DBS ARV levels consistent with ongoing ARV use during ATI are excluded from this analysis.

**Figure 6:**
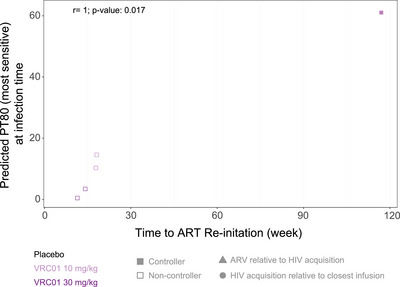
Scatter plot of time to ART initiation and predicted serum neutralization 80% inhibitory dilution titre (PT80) for the most sensitive (to VRC01) viral isolate. Five VRC01 recipients who completed ATI without ARV use are included, while placebo recipients (i.e. those who did not receive VRC01 in the AMP trial) are not included. Some participants acquired multiple viral isolates with varying degrees of VRC01 neutralization sensitivity. The PT80 of the most VRC01‐sensitive acquired isolate is reflected here.

AMP ATI participants have linked data from AMP and we compared non‐controllers and controllers by features of participants’ baseline HIV acquisition characteristics, including first positive VL (copies/ml), modelled VL kinetics (set point, peak and 3‐month average) [59], estimated time of HIV acquisition relative to closest VRC01/placebo infusion and time from estimated HIV acquisition to ART initiation (Figures 5 and S1). Additionally, we compared viral isolate characteristics associated with prevention efficacy in AMP [49, 58, 60], including IC80 to VRC01, VRC01 epitope distance and physiochemical‐weighted Hamming distance (Figures 6 and S2). No statistically significant differences were identified between controller and non‐controller participants in these parameters. In an analysis restricted to VRC01 recipients who initiated ATI (*n* = 5), a positive correlation between the PT80 of the most VRC01‐sensitive isolate and time to ART reinitiation was observed (*p* = 0.017).

## 4 DISCUSSION

Most ATIs conducted for research have been among men who have sex with men (MSM) in the global North [61, 62, 63]. Fewer ATIs have been conducted among women in Africa, reflecting the longstanding underrepresentation of women in HIV cure research [61, 63], the limited number of ATIs conducted in Africa and other regions with generalized HIV epidemics, and ongoing concerns about ATI, including potential transmission of HIV during pregnancy or nursing while off ART, potential disruption of public health messaging regarding ART adherence and/or potential disruption of participants’ access to ART through public health systems.

The rare ATIs conducted among women in Africa have yielded intriguing observations suggesting unique dynamics of viral control among African women compared to observations in Western MSM, and potentially compared to those in Western women [64, 65, 66]. Our work reinforces the observation of potentially more frequent PTC among African women, affirms the safety of ATIs conducted in this context, illuminates African women's motivations and barriers to ATI participation, and demonstrates a path for successful ATI conduct with southern African women going forward.

Among the 11 evaluable participants, we observed two participants (18%) with virologic control. In one participant who received VRC01 proximate to HIV acquisition, durable control of the virus to very low levels (undetectable or <200 copies/ml) persisted for 2 years. While infrequent in this cohort, this outcome of viral suppression with stable CD4 counts and no related AEs is essentially what functional HIV cure strategies are pursuing. This frequency of control aligns with that observed in other cohorts of early treated individuals [36] and in several small trials of bnAbs administered immediately prior to or during ATI [7, 8, 35, 67]. Our data also demonstrate the usefulness of a control group in ATIs to limit potential misattribution of observed control; in African women, delayed rebound and PTC is observed comparatively frequently, yet mechanisms remain incompletely understood and there are no robust historical controls.

Our study was small and we found no convincing links with potential predictors of viral rebound. Even in this cohort monitored with approximately 2 years of monthly pre‐acquisition HIV diagnostics, and after applying advanced modelling methods to supplement this frequent sample collection [59], the heterogeneity of HIV transmission and early viral dynamics limits our ability to fully characterize potential peri‐acquisition biomarkers predictive of later ART‐free virologic control. We identified no clear associations of VRC01 versus placebo receipt, pre‐ART viral dynamics or characteristics, or baseline HIV characteristics with controller versus non‐controller status. This may be attributable to our small population with considerable variability in these parameters and to other factors, including the antibody itself; for example, VRC01 may have had insufficient neutralization potency against our participants’ transmitted isolates, poor Fc effector functionality, or other characteristics limiting its formation of effective immune complexes. However, we did observe a significant positive association between the PT80 of the most‐sensitive acquired isolate and time off ART (on ATI) among the five VRC01 recipients who initiated ATI, consistent with VRC01 in sufficient concentration at acquisition and sufficient potency against one acquired isolate (the most VRC01‐sensitive) to bind HIV and potentially form immune complexes in the VRC01‐recipient controller. Additional immunologic and virologic assays are ongoing and may further our understanding of other associations with ART‐free virus control; evaluating different bnAbs, including those administered in early HIV, with and without ART, could further elucidate bnAbs’ potential role in later virologic control.

Engagement of African women in studies that interrupt the standard of HIV treatment, and potentially increase the chance of HIV transmission to partners and/or children, necessitates authentic engagement and careful consideration by participants, their communities and the scientific leadership where the research is conducted. Researchers should ensure that such engagement is accessible and incorporates opportunities for stakeholder discussion and feedback into trial design. Pre‐AMP ATI multi‐stakeholder engagement informed risk mitigation strategies and provided forums to address questions.

The potential risks of ATIs are well‐documented [56, 57, 68, 69]. We observed no HIV transmissions, SAEs, HIV‐related AEs or pregnancies. However, frequent screening revealed several STIs on‐study, some of which may have been reactivations of pre‐existing infections. Approximately one‐third of STIs were diagnosed during ATI, despite barrier protection eligibility requirements and transmission prevention counselling at each visit. This STI incidence supports ongoing attention to the potential of HIV transmission and may reflect challenges women face negotiating barrier protection use in their sexual relationships [70], though the absence of reported negative outcomes supports this study's approach to offer counselling, treatment and reassessment rather than mandatory discontinuation of participants with STIs.

The frequency of STI diagnoses also highlights the importance and challenge of incorporating approaches to assess and mitigate potential HIV transmission in partnership with participants while balancing respect for participant privacy and autonomy. Mindful of the latter, the AMP ATI did not require participants to include sexual partners in screening, informed consent or other trial procedures, an approach since validated in a similar population [48]. Instead, AMP ATI staff relied on a multi‐pronged informed consent process, counselling and their pre‐existing relationship with ATI participants who contributed to the earlier AMP Study. Often, this existing relationship enhanced communication and screening throughout AMP ATI informed consent and eligibility assessment. Still, approximately half of the participants reported an STI during ATI.

The existing relationship between participants and CRS staff may have mitigated risk in other ways, however. For example, AMP ATI participant safety was enhanced by outstanding retention. Missed visits are common in long trials with demanding visit schedules, but weekly or biweekly visits are important for frequent VL monitoring and other ATI risk mitigation strategies. The intensive schedule, study risks and other elements of the study design were well‐conveyed throughout screening, which was largely conducted by CRS staff with whom participants had a longstanding relationship preceding ATI. As ATIs continue across distinct cultural contexts, including among participants without pre‐existing relationships with CRSs, studies will need well‐considered approaches that both respect participant autonomy and provide support and protection strategies to identified partners [71].

## 5 CONCLUSIONS

This trial underscores the importance of ATI conduct across cultural contexts and the reality that they can be done safely and well with women in Africa. Elements that contributed to the successful development and implementation of the AMP ATI in southern Africa included authentic multi‐stakeholder engagement during design, development and implementation; robust informed consent processes, including participant‐centred decision questionnaires repeated throughout the trial; and close monitoring with frequent counselling. Going forward, the AMP ATI will further evaluate peri‐acquisition data from AMP, linked immunoassay data from AMP and the AMP ATI, and additional immunologic and virologic ATI data to better understand potential contributions of early ART with or without bnAbs to the course of HIV among southern African women, a unique and essential stakeholder population in the pursuit of ART‐free alternatives for sustained HIV suppression.

## COMPETING INTERESTS

The authors declare no conflict of interest.

## AUTHORS’ CONTRIBUTIONS

SK, PA, CO, LG, CK, GB, KB, JL, MA, ST, RT, LS‐T, AD, IMS, LC, GG, ACD and KB designed the clinical protocol. FL, SD, JM, MCH, C‐AM and WB recruited participants and generated clinical data. P‐CY, DG and ADC performed statistical and data analyses and directly accessed and verified the underlying data reported in the manuscript. SK, ADC and KB drafted the initial manuscript. All authors helped with revisions and provided final approval for it to be published.

## FUNDING

This study was supported by HVTN Statistical and Data Management Center [CDMC], Grant UM1 AI068635. HVTN Leadership and Operations Center, Grant UM1AI068614. HPTN Leadership and Operations Center, Grant UM1AI068619.

## ETHICS APPROVAL AND PATIENT CONSENT

Each clinical research CRS's Ethics Board reviewed and approved the study protocol and procedures. All participants demonstrated an understanding of the protocol and provided written informed consent prior to enrolment.

## Supporting information




**Figure S1. Viral Load Metrics**. (A) First positive viral load observed in the parent AMP trial HVTN 703/HPTN 081. Model estimated metrics (B) set point, (C) peak and (D) 3‐month average, based on pre‐ART viral load data from the parent AMP trial [59]. Two participants with DBS ARV levels consistent with ongoing ARV use during ATI are excluded from this analysis. Participant 805‐131856 (indicated with *) did not have enough data to fit the models in B–D. Filled squares indicate controller, open squares indicate non‐controller. Placebo (black), VRC01 30 mg/kg (dark purple) and VRC01 10 mg/kg (light purple).
**Figure S2. IC80 values of primary viral isolates**. (A) Epitope Distance (B) Physicochemical distance (C) Geometric mean IC80 was right censored at 100 µg/ml. Two participants with DBS ARV levels consistent with ongoing ARV use during ATI are excluded from this analysis. Participants 805‐861376, 805‐907560, 805‐370244, 805‐131856, and 805‐266234 each had two primary viral isolates. The two isolates for 805‐861378 indicated with * have values ≥100. Metrics in panels A and B are from [60] and C is from [49]. Filled squares indicate controller, open squares indicate non‐controller. Placebo (black), VRC01 30 mg/kg (dark purple) and VRC01 10 mg/kg (light purple).

## Data Availability

The data generated in this study are provided at https://dataverse.harvard.edu/dataverse/hvtn805‐hptn093. All individual participant data have been de‐identified.
