# Supplementary material for: Analytical treatment interruption among women with HIV in southern Africa who received VRC01 or placebo in the Antibody Mediated Prevention Study: ATI stakeholder engagement, implementation and early clinical data
Source: J Int AIDS Soc. 2025 Jun 3;28(6):e26495. doi: 10.1002/jia2.26495 (PMC12134397; doi:10.1002/jia2.26495)
Supplement: Supplementary file 1 — Figure S1. Viral Load Metrics. (A) First positive viral load observed in the parent AMP trial HVTN 703/HPTN 081. Model estimated metrics (B) set point, (C) peak and (D) 3‐month average, based on pre‐ART viral load data from the parent AMP trial [59]. Two participants with DBS ARV levels consistent with ongoing ARV use during ATI are excluded from this analysis. Participant 805‐131856 (indicated with *) did not have enough data to fit the models in B–D. Filled squares indicate controller, open squares indicate non‐controller. Placebo (black), VRC01 30 mg/kg (dark purple) and VRC01 10 mg/kg (light purple). Figure S2. IC80 values of primary viral isolates. (A) Epitope Distance (B) Physicochemical distance (C) Geometric mean IC80 was right censored at 100 µg/ml. Two participants with DBS ARV levels consistent with ongoing ARV use during ATI are excluded from this analysis. Participants 805‐861376, 805‐907560, 805‐370244, 805‐131856, and 805‐266234 each had two primary viral isolates. The two isolates for 805‐861378 indicated with * have values ≥100. Metrics in panels A and B are from [60] and C is from [49]. Filled squares indicate controller, open squares indicate non‐controller. Placebo (black), VRC01 30 mg/kg (dark purple) and VRC01 10 mg/kg (light purple). [file JIA2-28-e26495-s001.docx]

Supplemental Figures


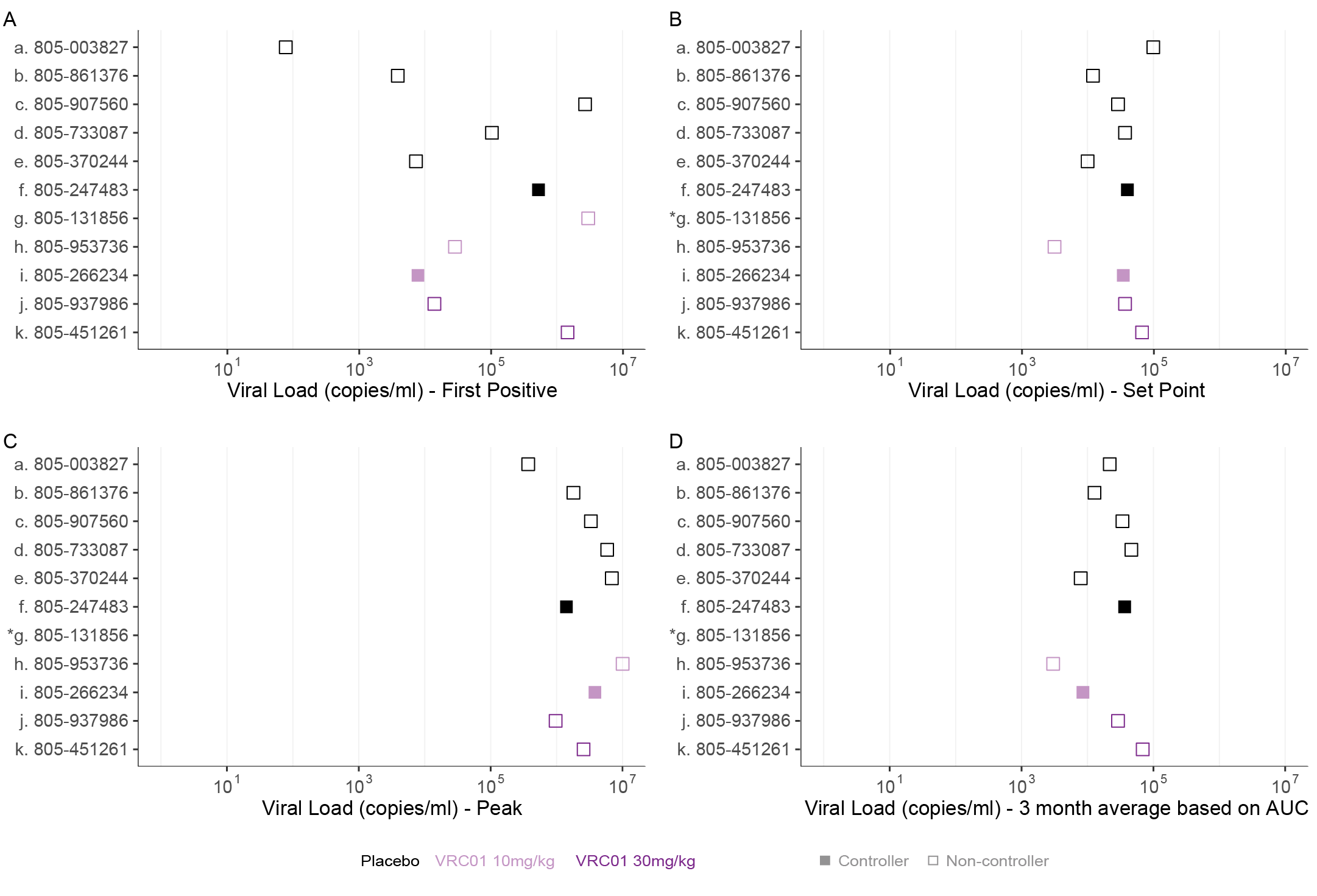


**Figure** **S1. Viral Load Metrics.** (A) First positive viral load observed in the parent AMP trial HVTN 703/HPTN 081. Model estimated metrics (B) set point, (C) peak and (D) 3-month average, based on pre-ART viral load data from the parent AMP trial^59^. Two participants with DBS ARV levels consistent with ongoing ARV use during ATI are excluded from this analysis. Participant 805-131856 (indicated with *) did not have enough data to fit the models in B-D. Filled squares indicate controller, open squares indicate non-controller. Placebo (black), VRC01 30mg/kg (dark purple) and VRC01 10mg/kg (light purple).


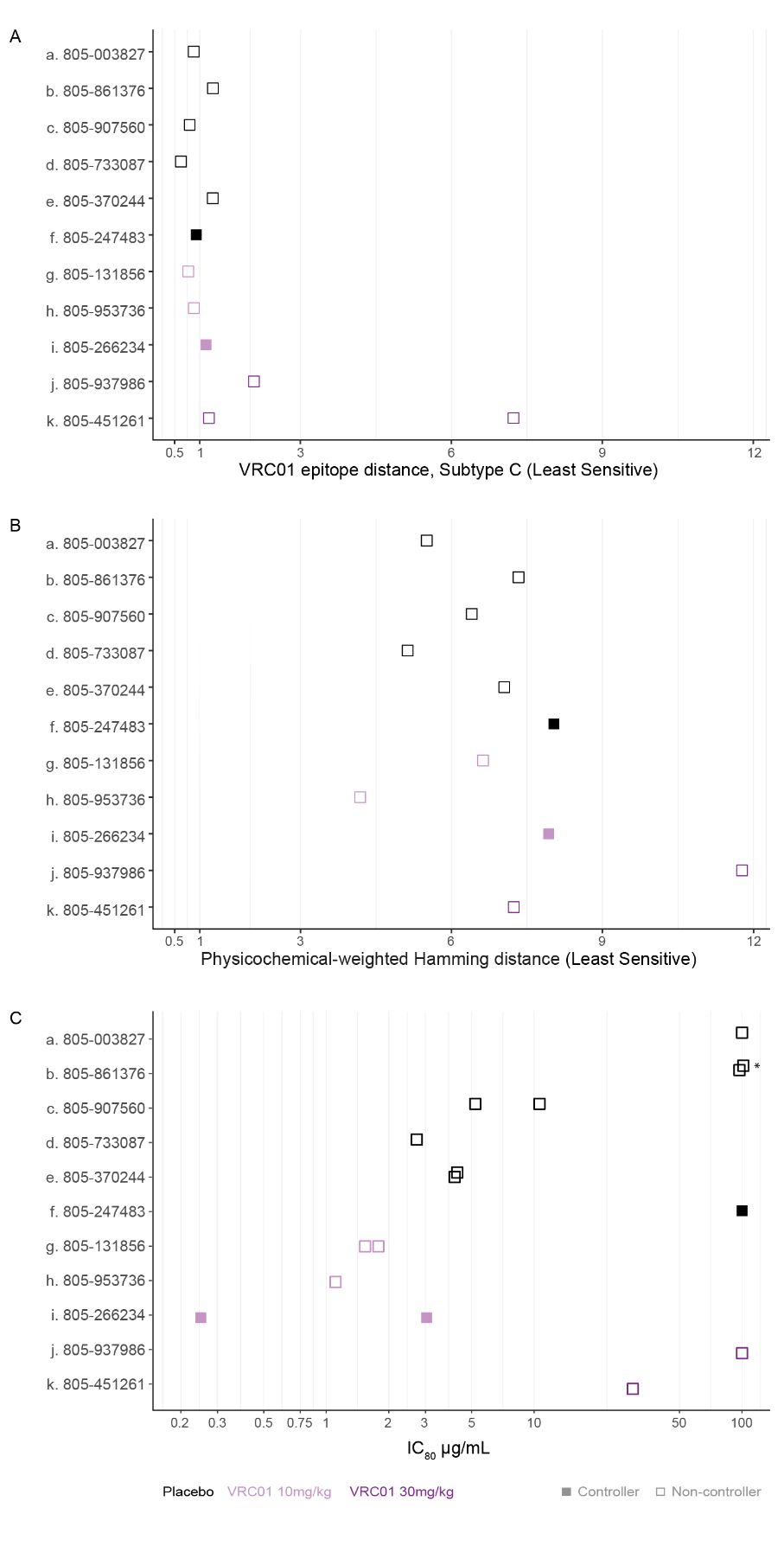


**Figure S2. IC80 values of primary viral isolates.** (A) Epitope Distance (B) Physicochemical distance (C) Geometric mean IC80 was right censored at 100 μg/ml. Two participants with DBS ARV levels consistent with ongoing ARV use during ATI are excluded from this analysis. Participants 805-861376, 805-907560, 805-370244, 805-131856, and 805-266234 each had two primary viral isolates. The two isolates for 805-861378 indicated with * have values ≥100. Metrics in panels A and B are from^60^ and C is from^49^. Filled squares indicate controller, open squares indicate non-controller. Placebo (black), VRC01 30mg/kg (dark purple) and VRC01 10mg/kg (light purple).
